# Fertility trends and comparisons in a historical cohort of US women with primary infertility

**DOI:** 10.1186/s12978-021-01313-6

**Published:** 2022-01-18

**Authors:** Emily Sadecki, Amy Weaver, Yulian Zhao, Elizabeth A. Stewart, Alessandra J. Ainsworth

**Affiliations:** 1 College of Medicine and Science, Mayo Clinic, 200 1st Street NW, Rochester, MN USA; 2 Division of Clinical Trials and Biostatistics, Mayo Clinic, Rochester, MN USA; 3 Division of Reproductive Endocrinology and Infertility, Department of Obstetrics and Gynecology, Mayo Clinic, 200 1st Street NW, Rochester, MN USA

**Keywords:** Primary infertility, Fertility, Epidemiology, Public health

## Abstract

**Background:**

There is growing interest in long-term outcomes following infertility and infertility treatment. However, there are few detailed longitudinal cohorts available for this work. This study aimed to assemble a historical cohort of women with primary infertility and age-matched controls to evaluate fertility trends, sequelae, and sociodemographic differences. Described here are cohort group characteristics and associated reproductive trends over time.

**Methods:**

A population-based historical cohort was created using the Rochester Epidemiology Project (REP) record-linkage system (Olmsted County, MN). The cohort included women aged 18–50 with a diagnosis of primary infertility between January 1, 1980, and December 31, 1999. As part of a case–control study, we identified 1:1 age-matched female controls from the same community and era.

**Results:**

A total of 1001 women with primary infertility and 1001 age-matched controls were identified. The women with primary infertility were significantly more likely to be married, college educated, use barrier contraception, and non-smokers compared to age-matched controls. The incidence of primary infertility increased from 14 to 20 per 10,000 person years from 1980–1985 to 1995–1999. Ovulatory dysfunction and unexplained infertility were the most common causes of primary infertility and clomiphene was the most widely used fertility medication. Rates of in vitro fertilization (IVF) increased from 1.8% during 1980–1985 to 26.0% during 1995–1999.

**Conclusion:**

Women with primary infertility were found to have unique sociodemographic characteristics compared to age-matched control women, which is consistent with previous research. The incidence of diagnosed primary infertility increased from 1980 to 1999, as did use of IVF.

**Supplementary Information:**

The online version contains supplementary material available at 10.1186/s12978-021-01313-6.

## Plain language summary

This study aimed to assemble a historic cohort of women with primary infertility and age-matched control women. The cohort included 1001 women with primary infertility diagnosed between 1980 and 1999 and 1001 age-matched controls from the same community and era. This cohort demonstrated baseline differences between the primary infertility and control groups, including differences in marital status, education, use of barrier contraception and smoking status. Additionally, the cohort showed an increased incidence in diagnosis of primary infertility from 1980 to 1999. Creation of this cohort will enable future research focused on long-term outcomes following primary infertility diagnosis and treatment.

**Supplementary Information:**

The online version contains supplementary material available at 10.1186/s12978-021-01313-6.

## Background

Infertility prevention, detection, management, and long-term follow-up are areas of increasing public health interest [1, 2]. Infertility impacts a substantial portion of the United States—roughly 15 percent of women, with around 12 percent having received infertility services [3, 4]. National databases have been developed to track infertility rates and treatment outcomes. There are two prominent databases in the United States. First, the National Survey for Family Growth (NSFG) periodically conducts population-level surveys of women aged 15 to 49, which includes questions about fertility status. Originally the NSFG only included married women, but it expanded to include non-married women in 1982 [5]. While the NSFG provides a population-level snapshot, it relies on self-report and does not include granular detail about infertility diagnosis or treatment. An additional US-based resource comes from a partnership between the Centers for Disease Control and Prevention (CDC) and the Society for Assisted Reproductive Technology (SART). Since 1995, they have reported annual outcomes from fertility clinics across the United States [3]. This includes more specific data about infertility diagnosis and treatment but does not include the population of women who do not seek care in specialty fertility clinics. Internationally, there are other noteworthy population-based registries. The most notable is the Danish cohort study developed from national registry data. The Danish study shows the potential value of a population-based cohort, both for investigating potential risk factors for infertility [6, 7] and long-term outcomes for both the infertile individual [8–10] and offspring [11, 12]. The Danish cohort began including outpatient medical contacts in 1995, limiting to a degree, the current availability of long-term follow-up past middle age.

Interest in the long-term health implications of infertility is growing [13–15], while the tools to properly evaluate these outcomes are lacking. Infertility is often multifactorial, with both male and female factors found to impact the likelihood of successful conception. A complete understanding of infertility likely reflects a combination of genetic causes, environmental impacts, and underlying disruption of hormonal and endocrine homeostasis [16]. The impact of chronic disease on fertility is well-documented due to its relevance to the treatment of infertility and associated obstetrical outcomes [16]. The corollary—impact of infertility on chronic disease—is also important because it could have implications for future screening and healthcare beyond a woman’s reproductive years. Initial studies have shown that a prior diagnosis of infertility is associated with an increased risk of several conditions including mental health disorders, diabetes, renal disease, cerebrovascular, and cardiovascular disease [14, 15, 17–19]. These prior studies vary in methodology of infertility identification, ranging from patients who presented for any fertility evaluation to those who were seen by a fertility subspecialist or pursued assisted reproductive technologies (ART) [17]. A comprehensive evaluation of infertility-associated long-term outcomes requires investigation of potential confounders such as type of infertility, type of fertility treatment, and subsequent parity with use of a population-level cohort.

Given the lack of longitudinal cohorts in the United States, this study aimed to assemble a historical cohort of women with primary infertility to allow for subsequent evaluation of reproductive and long-term health outcomes. Herein, the methodology used to create the **M**ayo Clinic **P**rimary **I**nfertility **C**ohort (MPIC) is described with initial descriptive findings presented on the overall incidence and changes in fertility diagnoses and treatment from 1980 to 1999. Importantly, an age-matched cohort of female controls from the same community was assembled which will allow us to assess the impact of infertility, and not specifically infertility treatment, on long-term outcomes in future studies. Conversely, women without a male partner (either single or in a same sex relationship) or a partner with prior vasectomy were included to evaluate the impact of fertility treatment, without known underlying infertility, on long-term outcomes in future studies. In this study we report on the incidence of primary infertility using the MPIC (cases) and the results of a case–control study to evaluate sociodemographic differences.

## Methods

### Cohort identification

Women aged 18–50 with a diagnosis of infertility from January 1, 1980 through December 31, 1999 were identified using the Rochester Epidemiology Project (REP) medical records-linkage system [20]. The REP includes complete medical records for all medical providers in Olmsted County, Minnesota and provides an opportunity for longitudinal retrospective review of residents in Olmsted County. Additional details of the REP and of the Olmsted County population have been previously described [21].

**Fig. 1:**
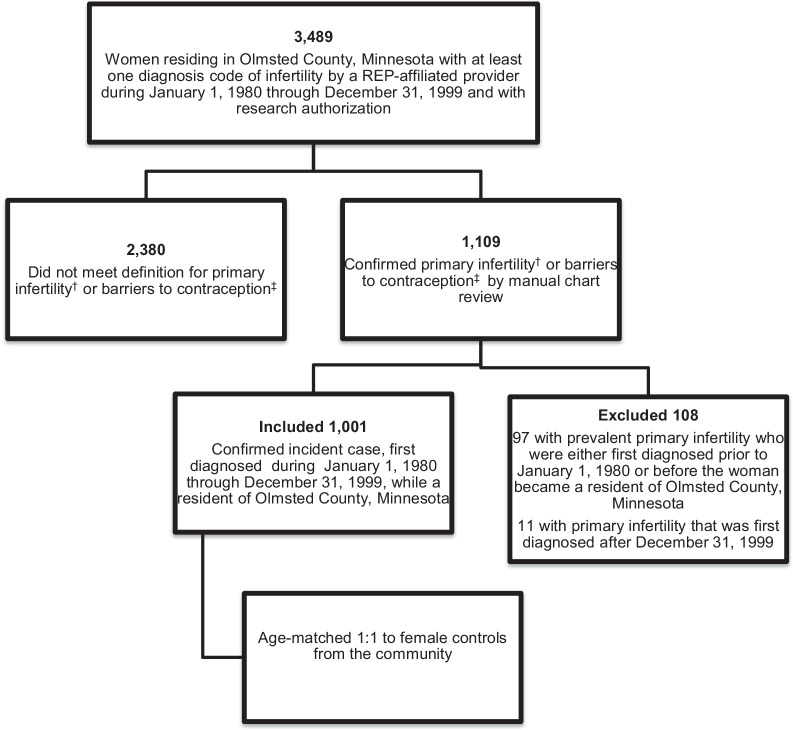
Mayo Primary Infertility Cohort (MPIC) flow chart

A total of 3489 women aged 18–50 with at least one diagnosis code of infertility and who had research authorization were identified through the REP diagnostic indices (Fig. 1). This study focused on women who were first diagnosed with primary infertility from January 1, 1980 to December 31, 1999, while a resident of Olmsted County, to allow for adequate time to assess long-term outcomes in subsequent publications. Secondary infertility was not included to reduce potential confounding variables, such as effects of prior treatment and prior parity on long-term outcomes. Primary infertility was defined as an inability to conceive after 12 months of attempted conception in women aged < 35 and after 6 months of attempted conception in women ≥ 35 [22]. Women with known barriers to conception including same-sex couples, women pursuing single parenting with donor sperm, and male partners with prior vasectomy were also included to allow for future study on the impact of fertility treatment, without the compounded effect of underlying infertility. A total of 1001 women were identified who had a diagnosis of primary infertility confirmed by chart review that met the criteria described above. The clinical note dated at their first evaluation for primary infertility was defined as the “index date”.

### Matched controls

For the case–control study, each confirmed woman with primary infertility was 1:1 age-matched (± 1 y) to a referent/control woman randomly selected from the women residing in Olmsted County at the time of the index date who had not been diagnosed with primary infertility prior to the index date. Control women were identified using a matching algorithm available through the REP that utilizes information on each patient’s residency history at the time of their medical visits. Upon reviewing the medical records, potential controls identified with infertility issues prior to the index date were replaced.

### Data collection

The medical records of the women with confirmed primary infertility and their matched controls were manually reviewed between September 2019 and November 2020. Three individuals (AA, ES and LKR) completed all chart reviews. To ensure consistency and refine the data collection tool, AA reviewed the first 50 charts in duplicate, with ES and LKR. Any charts with unclear data were subsequently reviewed by AA. Data collected included baseline demographics, gynecologic history, past medical and psychiatric history, fertility, and obstetrical history. Baseline demographics as of the index date were collected retrospectively and included race, marital status, years of education completed, tobacco use (current, prior, or none) and body mass index (BMI). The length of attempted conception in months prior to diagnosis was recorded along with type of infertility. Type of infertility could include multiple diagnoses and included ovulatory dysfunction, male factor, tubal factor, uterine factor, or unexplained infertility. Women with a history of ovulatory dysfunction were further classified as having amenorrhea, polycystic ovary syndrome, oligo-ovulation, diminished ovarian reserve, or hypothalamic hypogonadism [23]. Male factor infertility was further classified as abnormal semen parameters or absence of male partner for same-sex female couples or women pursuing single parenting. Women were classified as unexplained infertility if they had normal evaluations without evidence of other causes of infertility.

Gynecologic history was collected by chart review and included age of menarche, whether they had regular menstrual cycles at index date, and if they had previously used contraception. If they reported a history of contraceptive use, the type(s) was documented as combined estrogen and progesterone methods, progestin only methods, intrauterine devices and type, or barrier methods.

Fertility and obstetrical histories were collected by chart review. The type of fertility treatment, if any, was reviewed and included use of clomiphene, letrozole, gonadotropins, or in vitro fertilization (IVF). The number of IVF cycles was recorded and whether oral medications or gonadotropins were used for greater than or less than one year prior to a positive pregnancy test were recorded. The year of first pregnancy and use of fertility medications (yes or no) prior to this first pregnancy was also recorded.

### Statistical methods

Age-specific incidence rates of primary infertility in Olmsted County during 1980–1999 were calculated; the numerator was the number of persons with an incident diagnosis of primary infertility, and the Olmsted County denominator was obtained from the REP census for women aged 18–45 [24]. Rates were age-adjusted to the total population structure of the United States in 2010, since this was the most current population structure available. The 95% confidence intervals (CIs) for the rates were calculated assuming a Poisson error distribution. The incidence rates between age groups or calendar periods can be statistically compared by examining the overlap of the confidence intervals for any two rates; non-overlapping confidence intervals indicate that the rates are significantly different at the 0.05 level, however overlapping confidence intervals do not necessarily indicate that the rates are not significantly different at the 0.05 level.

Data were summarized using standard descriptive statistics: frequency counts and percentages for categorical variables and mean (SD, standard deviation) for normally distributed continuous variables or median (IQR, interquartile range) for skewed continuous variables. Distributions of continuous variables were assessed graphically for normality and skewness. Consistent with the case–control study design, patient characteristics at the time of the index date were each evaluated for an association with primary infertility status (yes vs. no) based on fitting univariable conditional logistic regression models. A full multivariable conditional logistic regression model was fit including BMI and the following dichotomized (yes vs. no or not documented) variables: caucasian, hispanic, married, college graduate or beyond, ever smoker, regular menstrual cycles, and contraceptive use. Associations were summarized by reporting odds ratios (OR) and corresponding 95% confidence intervals (CI) estimated by the models. Odds ratios represent the ratio of the odds of exposure among cases with primary infertility relative the odds of exposure among controls. For BMI, the odds was per a 5 kg/m^2^ increase in BMI and the median BMI in each group was imputed for those missing BMI prior to fitting the multivariable model. All calculated p-values were two-sided. Data was analyzed using SAS version 9.4 statistical software (SAS Institute, NC; Cary, NC).

## Results

Among the 1001 women, aged 18–50 years, identified as an incident case with primary infertility from 1980 through 1999, the mean age at diagnosis was 29.2 years (SD, 4.4) with a range of 18.4–45.9 years. The index date ranged from 1980 to 1999 with the year of first primary infertility diagnosis distributed as follows: 221 (22.1%) in 1980–1984, 234 (23.4%) in 1985–1989, 247 (24.7%) in 1990–1994, and 299 (29.9%) in 1995–1999. Overall, for women aged 18–45 the age-adjusted incidence of primary infertility in Olmsted County during this period was 16.8 (95% CI 15.7–17.8) per 10,000 person-years.

**Fig. 2:**
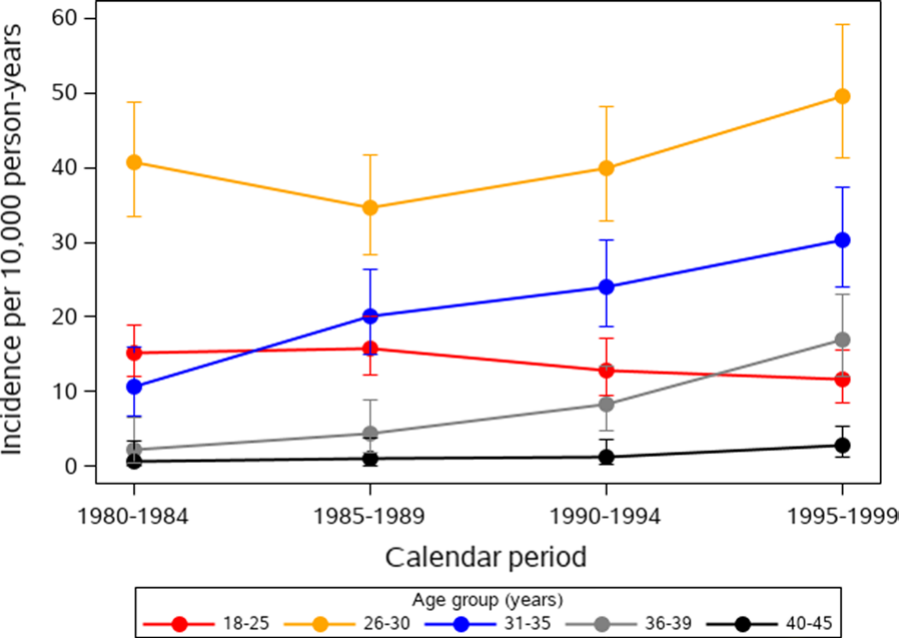
Incidence of primary infertility diagnosis per 10,000 person-years in Olmsted County, Minnesota, stratified by calendar period and age group. Error bars represent 95% confidence intervals

The age-specific incidence rate (per 10,000 person-years) was highest among those in the 26–30 age group at 40.8 (95% CI 37.1–44.7), followed by 22.1 (95% CI 19.2–25.0) in the 31–35 age group, 14.0 (95% CI 12.3–15.9) in the 18–25 age group, 9.1 (95% CI 7.0–11.5) in the 36–39 age group, and 1.6 (95% CI 0.8–2.6) in the 40–45 age group. The overall age-adjusted incidence has gradually increased over time with rates of 14.0 (95% CI 12.1–15.9), 15.1 (95% CI 13.1–17.0), 16.4 (95% CI 14.4–18.5), and 20.4 (95% CI 18.0–22.7) in 1980–84, 1985–89, 1990–94, and 1995–1999, respectively. Figure 2 presents the age-specific rates by 5 year calendar periods.

Demographic, social, and reproductive characteristics at the time of the index date were compared between the women with primary infertility and age-matched controls (Table 1). Among those with documented information on race or ethnicity, both groups were primarily Caucasian (93.8% [797/850] of women with primary infertility and 93.9% [761/810] of controls) and not Hispanic or Latino (96.7% [726/751] of the women with primary infertility and 98.8% [722/731] of controls). Marital and education status differed significantly between the groups with the odds of being married women (OR 15.5, 95% CI 10.1–23.92) or a college graduate (OR 1.78, 95% CI 1.48–2.14) being higher among women with primary infertility compared to controls. The odds of ever smoking prior to the index date was lower for women with infertility (OR 0.54, 95% CI 0.45–0.66). The mean BMI was slightly lower among the women with primary infertility, but the difference was not clinically meaningful albeit statistically significant. Age at menarche was 13 (IQR, 12, 14) for both groups. The odds of having regular periods was also lower among women with primary infertility compared to controls (OR 0.49, 95% CI 0.40–0.61). Lastly, the odds of prior contraceptive use was higher among women with primary infertility compared to controls (OR 1.26, 95% CI 1.02–1.55), specifically for the use of barrier contraception (OR 1.76, 95% CI 1.27–2.43). Upon fitting a full multivariable model, all of the characteristics remained statistically significant except for BMI and prior contraceptive use (Table 1).

**Table 1:** Demographic, social and reproductive characteristics at the time of the index date of women diagnosed with primary infertility between 1980 and 1999 and age-matched female controls

Characteristic at the index date^†^	Primary infertility (N = 1001)	Controls (N = 1001)	Unadjusted OR (95% CI) from univariable models	Adjusted OR (95% CI) from a full multivariable model
Age (years)			–^§^	–^§^
Mean (SD)	29.2 (4.4)	29.2 (4.4)		
Range	(18.4–45.9)	(18.2–46.1)		
18–25	238 (23.8%)	232 (23.2%)		
26–30	455 (45.5%)	451 (45.1%)		
31–35	228 (22.8%)	241 (24.1%)		
36–40	72 (7.2%)	67 (6.7%)		
41–50	8 (0.8%)	10 (1.0%)		
Race				
Caucasian	797 (79.6%)	761 (76.0%)	1.24 (1.00, 1.53)	1.33 (1.00, 1.76)
Black or African American	11 (1.1%)	5 (0.5%)		
Asian	28 (2.8%)	23 (2.3%)		
Native Hawaiian or Pacific Islander	0 (0.0%)	1 (0.1%)		
American Indian or Alaskan Native	1 (0.1%)	6 (0.6%)		
Other	13 (1.3%)	14 (1.4%)		
Unknown or chose not to disclose	151 (15.1%)	191 (19.1%)		
Ethnicity				
Hispanic or Latino	25 (2.5%)	9 (0.9%)	3.00 (1.35, 6.68)	4.48 (1.69, 11.84)
Not Hispanic or Latino	726 (72.5%)	722 (72.1%)		
Unknown or chose not to disclose	250 (25.0%)	270 (27.0%)		
Marital Status				
Single	15 (1.5%)	337 (33.7%)		
Married	966 (96.5%)	646 (64.5%)	15.54 (10.10–23.92)	14.83 (9.51, 23.11)
Partnered (not married)	20 (2.0%)	10 (1.0%)		
Not documented	0 (0.0%)	8 (0.8%)		
Level of education				
Less than high school	12 (1.2%)	33 (3.3%)		
High school graduate	129 (12.9%)	201 (20.1%)		
Some college	274 (27.4%)	335 (33.5%)		
College (4-yr) graduate	340 (34.0%)	226 (22.6%)	1.78 (1.48, 2.14)	1.74 (1.37, 2.21)
Beyond college	159 (15.9%)	135 (13.5%)		
Not documented	87 (8.7%)	71 (7.1%)		
Tobacco use				
Current or Former	225 (22.5%)	356 (35.6%)	0.54 (0.45–0.66)	0.70 (0.55, 0.90)
Never	770 (76.9%)	632 (63.1%)		
Not documented	6 (0.6%)	13 (1.3%)		
BMI (kg/m^2^)				
Mean (SD)	24.1 (5.7)	24.8 (5.9)	0.90 (0.83, 0.97)	0.98 (0.89, 1.09)
Less than 18.5	49 (4.9%)	26 (2.6%)		
18.5–24.9	645 (64.4%)	617 (61.6%)		
25–29.9	179 (17.9%)	190 (19.0%)		
30–39.9	88 (8.8%)	118 (11.8%)		
40 or more	27 (2.7%)	28 (2.8%)		
Not documented	13 (1.3%)	22 (2.2%)		
Regular menstrual cycles				
Yes	706 (70.5%)	830 (82.9%)	0.49 (0.40, 0.61)	0.51 (0.39, 0.67)
No	295 (29.5%)	130 (13.0%)		
Not documented	0 (0.0%)	41 (4.1%)		
Contraceptive use prior to index date				
Yes	765 (76.4%)	723 (72.2%)	1.26 (1.02, 1.55)	0.99 (0.77, 1.29)
No	230 (23.0%)	249 (24.9%)		
Not documented	6 (0.6%)	29 (2.9%)		
Contraceptive use prior to index date				
Combined (estrogen/progesterone containing pills, patches, or inserts)	715 (71.4%)	675 (67.4%)	1.22 (1.00, 1.48)	
Progestin only	7 (0.7%)	15 (1.5%)	0.47 (0.19, 1.14)	
Intrauterine device	12 (1.2%)	19 (1.9%)	0.61 (0.29, 1.29)	
Implant (Nexplanon, Implanon)	4 (0.4%)	9 (0.9%)	0.44 (0.14, 1.44)	
Barrier	107 (10.7%)	63 (6.3%)	1.76 (1.27, 2.43)	

*CI* confidence interval, *BMI* body mass index, *OR* odds ratio

^†^ The index date for each matched pair (case and control) was defined as the date when the infertility case was first diagnosed with primary infertility

^‡^Associations were evaluated based on fitting univariable conditional logistic regression models (unadjusted results) and a full multivariable model (adjusted results). Odds ratios represent the ratio of the odds of exposure among cases with primary infertility relative the odds of exposure among controls, where exposure status for each of the variables was dichotomized (yes, no): caucasian, hispanic, married, college graduate or beyond, ever smoker, regular menstrual cycles, and contraceptive use (vs. all of the other levels for the variable combined, including not documented). For BMI, the odds was per a 5 kg/m^2^ increase in BMI

^§^Age was not statistically compared as each infertility case was 1:1 age-matched (± 1 y) to a female control

Table 2 summarizes the infertility characteristics of the women with primary infertility. The median length of infertility was 16 months (IQR, 12–24 months) [16]. The primary infertility etiology was unexplained infertility (37.4%) followed by ovulatory dysfunction (31.6%) and male factor (4.6%). The least common etiologies of primary infertility included tubal factor (6.2%) and uterine factor (2.9%). The most common fertility treatment was clomiphene (58.7%), followed by gonadotropins (20.9%) and IVF (16.8%). Most women eventually became pregnant (70.3%). Of those that achieved pregnancy, 59.9% utilized at least one fertility medication. Infertility treatments were also analyzed by time period of diagnosis (Additional file 1: Fig. S1). Of the three treatment options captured among the women with primary infertility, 35.3% used none, 40.6% used just one, 16.7% used two and 74.9% used all three treatment types. Clomiphene use remained relatively stable at 54.1% among those diagnosed between 1980 and 1984 versus 59.7% among those diagnosed between 1995 and 1999. Gonadotropin use increased from 4.6% (1980–1984) to 30.0% (1999–1994), then decreased to 22.9% (1995–1999). IVF use steadily increased from 1.8% (1980–1984) to 26.0% (1995–1999).

**Table 2:** Infertility characteristics and pregnancy outcomes in the Mayo Primary Infertility Cohort

Characteristic	Total (N = 1001)
Length of infertility (months)	
No. available	960
Median (IQR)	16 (12, 24)
Type of Infertility^†^	
Ovulatory Dysfunction	316 (31.6%)
Amenorrhea	22
Oligo-ovulation	239
PCOS	41
Hypothalamic hypogonadism	10
Diminished ovarian reserve	9
Male factor	246 (24.6%)
Absence of a male partner	14
Abnormal semen parameters	231
Tubal factor	62 (6.2%)
Uterine	29 (2.9%)
Endometriosis	104 (10.4%)
Unexplained	374 (37.4%)
Fertility medications used prior to documented pregnancy^‡^	
Clomiphene	
No	413 (41.3%)
< 1 year use	529 (52.8%)
> 1 year use	59 (5.9%)
Gonadotropin	
No	792 (79.1%)
< 1 year use	201 (20.1%)
> 1 year use	8 (0.8%)
Letrozole	0 (0%)
IVF	168 (16.8%)
Ever pregnant	
No	297 (29.7%)
Yes, with fertility medications to achieve first pregnancy	422 (42.2%)
Yes, without fertility medications to achieve first pregnancy	282 (28.2%)

^†^Among the 1001 women and considering the six listed types of infertility, 883 had a single type of infertility, 107 had two of the types, 10 had three types and 1 had 4 types

^‡^Among the 1001 women, 353 used none of the four listed fertility medications, 406 used one option, 167 used two options and 75 used three options

## Discussion

The MPIC represents a unique and well-defined US population-based cohort with primary infertility along with age-matched controls. The MPIC highlights trends in reproduction and fertility care in the US from 1980–1999. In this population, the incidence of primary infertility increased from 14.0 (1980–1984) to 20.0 (1995–1999) per 10,000 person-years with increasing rates of infertility across all age groups, except those aged 18–25 whose rates of primary infertility decreased over time. These findings correlate with trends in reproduction during this period, such as delayed childbearing and associated increases in infertility [2]. Our study reflects a notable increase in rates of infertility and infertility care after 1989 which likely reflects the increase in awareness, acceptance, and access to advanced fertility treatments after the first successful live birth following IVF in 1981 [25]. Similarly, this trend is also reflected in rates of IVF treatments which increased from 1.8% in women diagnosed during 1980–1984 to 12.4% in women diagnosed during 1985–1989. The Danish cohort study showed similar trends in treatment usage for clomiphene and gonadotrophins during the overlapping time periods [26]. Although the trends found in our study correlate well with the history of reproductive care in the US, a direct comparison to the incidence and etiology of infertility in other studies is difficult given the lack of other US population-based cohorts reporting on incidence and differing methodologies of infertility identification [4, 27–29]. Our study is most similar to the Danish cohort study, for which crude numbers, but not incidence rates, are available [26]. To our knowledge, the MPIC is the only US population-based infertility cohort to-date.

While our cohort reports incidence during this time, multiple studies, including the NSFG, have captured prevalence of infertility through purposeful population sampling [27, 30]. The NSFG reported an increase in fecundity among married woman between 1982 (11%) and 2002 (15%) [28]. It is difficult to comment on our incidence rates in comparison to these prevalence data because of the differences in methodology.

In this study of the MPIC, we compared demographic, social, and reproductive characteristics of women with primary infertility to age-matched controls. Of note, at the time of the index date, the women with primary infertility were more likely to be married, have a 4 year college degree, and to be non-smokers compared to the controls. The marital status and education level findings reflect national survey data, which reported increased fertility service use among married women and those of higher educational level [28]. The increased infertility diagnoses among married women are likely related to married women being more likely to be actively trying to conceive, or to seek care for inability to conceive, rather than a reflection of difference in underlying fertility. Importantly, early survey data included only married couples and notably excluded individuals with infertility not meeting these criteria [31].

The increased use among women with higher educational status is likely multifactorial. A difference in educational level at index date could be related to delayed childbearing in women pursuing higher education, as increased age is associated with increased infertility [16]. Additionally, level of education is a proxy for socioeconomic status of women pursuing infertility treatment. Disparities in access to infertility services by income and education level has been demonstrated previously [32, 33], even in places with universal health insurance coverage [32]. Lastly, referral practices may also influence this difference. In one Australian study, patients from non-English speaking backgrounds and not having income assistance were more likely to be managed in primary care rather than be referred to a fertility clinic or specialist [34]. Given our population-based sample, this referral pattern is not likely to influence the patients included in the MPIC but may impact the timing and type of care they received.

An unexpected finding was the lower incidence of tobacco use in women with primary infertility, as smoking is a well-established risk factor for infertility [16, 35]. One potential explanation for this finding is the relationship between smoking status and education, with higher educational status being associated with lower rates of smoking [36–38]. As discussed previously, those with primary infertility were more likely to have a 4-year college degree when compared to control women, which may be a confounding variable in the difference in smoking status.

Finally, women with primary infertility had an increased use of barrier contraception when compared to controls, which is noteworthy, as barrier contraception is generally thought to be protective against infertility by preventing sexually transmitted diseases and subsequent tubal factor infertility [39]. Prior studies from this time period showed a higher rate of birth control use in women presenting for infertility care but may not have accounted for use of both barrier and pharmacologic contraception [40]. All of the above forementioned factors remained statistically significant in the full multivariable analysis except for use of any type of conception (any vs. none).

Compared to the most recent CDC ART report released in 2017, the MPIC differs in etiology of infertility, with higher rates of ovulatory dysfunction (31.6 vs 15%) and lower rates of tubal factor infertility (6.2 vs 11%). Overall, frequency of male factor, uterine factor, and endometriosis only differed slightly (24.6 vs 28%, 2.9 vs 6%, 10.4 vs 7%, respectively). These differences may reflect a difference in time period or highlight a potential difference in community-based samples (MPIC) versus fertility clinic reported data (CDC ART). Data from the 2000 CDC ART data also shows lower rates of ovulatory dysfunction across all age groups (2.8–7.0%) than in the MPIC [41], suggesting this difference may persist across time. A more contemporaneous Swedish cohort from 1861 to 1975, which included 2768 patients presenting to three separate obstetrics and gynecology departments, reported 42% as having ovulatory dysfunction [42]. This rate more closely resembles the results of the MPIC (31.6%) than the CDC data from fertility clinics either in 2000 or 2017. This could be related to the time period similarity between the Swedish cohort and the MPIC. Other cohorts do not comment on etiology of infertility to the granularity of the MPIC [10, 29, 43], making it difficult to draw further conclusions.

Beyond these initial descriptive analyses there are opportunities and plans to utilize the MPIC for investigation of long-term outcomes of infertility. Importantly, a cohort with this degree of patient-level detail is useful in a more comprehensive assessment of the impact of primary infertility on long-term health. As demonstrated here, the demographics, infertility specific diagnoses and care likely differ between population-based cohorts and subspecialty generated cohorts. Full exploration of long-term health consequences of infertility requires population-level assessment to yield generalizable results and reduce potential biases introduced when focusing on patients presenting subspecialty fertility clinics.

Overall, this study provides descriptive analysis of a well-defined US population-based cohort with primary infertility and matched controls. Comparing MPIC to other primary infertility registries as done here, outlines the importance of moving towards population-level studies regarding infertility and long-term health outcomes. While additional work is planned to use MPIC, there is also a need for additional US based population studies to more comprehensively represent the diversity of the US patient population. The MPIC is consistent in overall trends in infertility and known demographics of women with infertility, supporting its use as a representative sample of women with primary infertility in the US and highlighting population level trends in reproductive health and fertility care.

Strengths of the MPIC include the granular detail of data collected at index date via chart review and manual confirmation of primary infertility at time of chart review that does not rely on patient self-report or medical codes. Additionally, the historical timing of the cohort lends itself to answering questions about long-term health outcomes of primary infertility and infertility treatment. While the MPIC provides an opportunity for meaningful epidemiologic study, it does have limitations. Primarily, the population of Olmsted County, MN is rather homogenous with 99.1% of Olmsted County being Caucasian in 1970 and 90.3% in 2000 [44].This is consistent with our finding that 94% of our cases and controls with documented race were Caucasian. This limits the generalizability of our findings but provides an opportunity for future evaluation of identified trends in more racially diverse groups. A need for expanded representation of diverse racial groups is especially important as racial and ethnic disparities in accessing fertility treatment have also been recorded with non-Hispanic white women being more likely to access care than Black and Hispanic counterparts [28, 32, 33, 45]. Additionally, although the cohort aimed to develop a population-based sample of primary infertility, it could not capture women with primary infertility who did not seek medical evaluation or treatment for this condition.

## Conclusions

This study aimed to create a historic cohort of women with primary infertility and age-matched control women from a population-based sample. The primary goal of this initial analysis of the cohort is to describe demographic and treatment trends. This study found demographic differences in women with primary infertility compared to age-matched controls. Women with primary infertility were more likely be married, have a college-level education, use barrier contraception and less likely to be current users of tobacco. Rates of primary infertility increased from 14 to 20 per 10,000 person years across the study period. Ovulatory dysfunction and unexplained infertility were the most common causes of infertility and clomiphene was the most common type of fertility medication used. IVF use increased from 1.8 to 26.0% across the study period. Importantly, future use of this cohort will allow for better understanding of long-term outcomes of women with primary infertility and infertility treatment and provide an example from which other population-based infertility cohorts may be modeled and compared.

## Supplementary Information

**Additional file 1: Figure S1.** Percent of primary infertility cases who utilized clomiphene, gonadotropins and/or IVF treatment based on time period of diagnosis

## Data Availability

Data used and analyzed in this study are available from the corresponding author on reasonable request.

## Abbreviations

IVF: In vitro fertilization
NSFG: National Survey for Family Growth
CDC: Centers for Disease Control and Prevention
SART: Society for Assisted Reproductive Technology
ART: Assisted reproductive technology
MPIC: Mayo Clinic Primary Infertility Cohort
REP: Rochester Epidemiology Project
BMI: Body mass index
CI: Confidence interval
SD: Standard deviation
