# Supplementary figures and images for: Fertility trends and comparisons in a historical cohort of US women with primary infertility
Source: Reprod Health. 2022 Jan 18;19:13. doi: 10.1186/s12978-021-01313-6 (PMC8764822; doi:10.1186/s12978-021-01313-6)

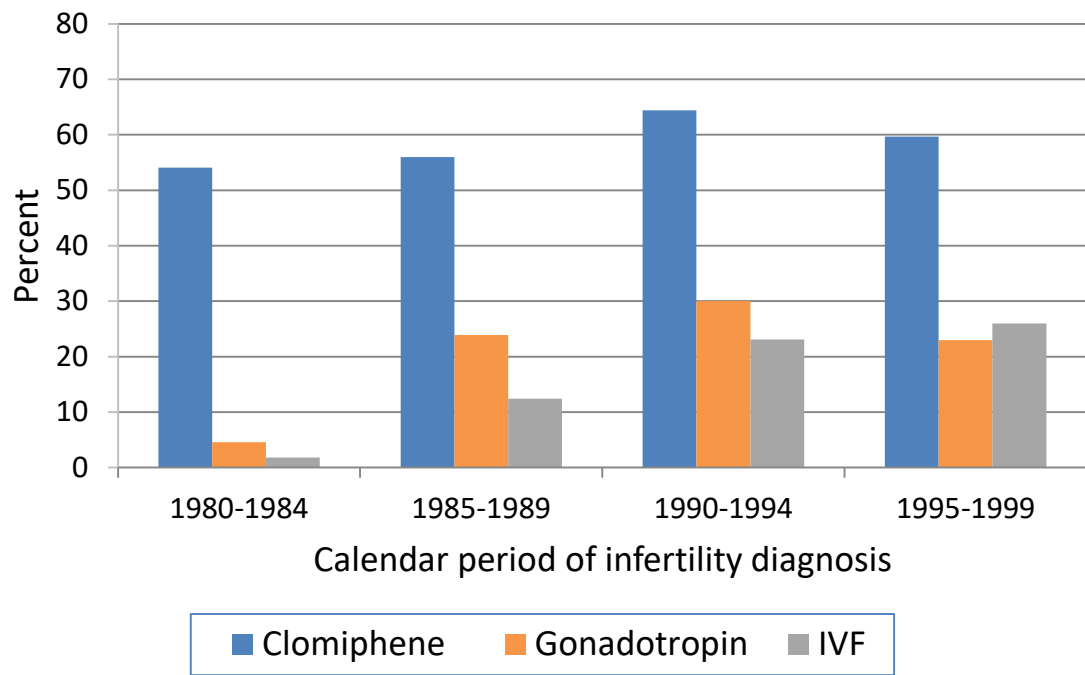

Supplement: Supplementary file 1 — Additional file 1: Figure S1. Percent of primary infertility cases who utilized clomiphene, gonadotropins and/or IVF treatment based on time period of diagnosis [file 12978_2021_1313_MOESM1_ESM.pdf]
